# Book Review: Speechless. My Recovery from Stroke

**DOI:** 10.3389/fpsyg.2017.01369

**Published:** 2017-08-09

**Authors:** Nereida Bueno-Guerra

**Affiliations:** ^1^Department of Psychology, Comillas Pontifical University Madrid, Spain; ^2^Department of Psychology and Clinical Psychobiology, University of Barcelona Barcelona, Spain; ^3^Department of Developmental and Comparative Psychology, Max Planck for Evolutionary Anthropology Leipzig, Germany

**Keywords:** stroke, cerebral hemorrhage, patient, caregiver, recovery, treatment, speech

*“When I read a new word-that is one I knew before the stroke- I knew what it meant intuitively. But although the new word was not lost it was not available to me. Concepts…would fade away like moonshine the moment I tried to pin them down with speech. It was a sensation so fleeting that I hardly knew it was there. The lack of speech ate right into my dealings with the outside world. It made me vulnerable, timid, defensive and shy. I hated being like that. I used to have opinions and laugh. People would say to me “You must feel very frustrated.” No, “frustration” definitely is not the word. I was aware of the fragmentation of my brain-the emotions, the body language and thoughts that were all cut off and out of my reach. To paraphrase Wordsworth, mine was a grief “too deep for tears.””* (p. 53, 111, 144, 150).

Stroke is one of the current primary causes of long-term disability and death (AHA, 2016). That is the reason why decoding its causes and looking for the best combination of treatments has turned into one of the main challenges of Medicine during the last decade. As a result, there is a vast array of useful handbooks for clinicians (e.g., the free downloadable handbook from the Canadian Partnership for Stroke Recovery's website^1^) or for neurologists (e.g., Hennerici et al., 2012) that provide meaningful knowledge on stroke. However, if the usefulness of those texts is invaluable, the objectivity about stroke clashes with the need for understanding at a subjective level specially by those who surround the patient everyday (relatives and caregivers) and even by those who turned to be new patients. “The stroke happened on the left temporo-medial arteria and therefore one of the derivate symptoms is aphasia.” OK. But what does this mean in the cinema when your affected-by-stroke-beloved-one listens to a dialogue? How does an aphasic patient experience comparing prices in a mall or reading the menu at a restaurant?

*Speechless* is a 170-paged autobiographical book written in 1994 by Jennifer Gordon, a patient recovered from a stroke. Since then, many others have come to join this specialized literature, such as the best-seller “My stroke of insight: a brain scientist's personal journey” (Taylor, 2008) or the pragmatic “After a stroke: 300 tips for making life easier” (Hutton, 2005). Thus, what makes Jennifer's work especially different? In the former case, we find a very educational piece of work, since the book is written by a neuroscientist who suffered a stroke and therefore experienced his brain impairment from a particular expert-like perspective. The latter case is a compendium of advices for the daily routine of a stroke patient. However, *Speechless* does not pursue any educational nor pragmatic goal. Her author just talks –shares–, as a diary, every single thought, difficulty and way to cope with her emotions, people around and a suddenly too new environment since the acute phase till the most recovered stages of her stroke. Therefore, the book structure is chronological. On the positive side, this allows to empathizing with the patient; advancing with her in the treatment and getting to know how dozens of daily troublesome activities that normative people might have not found as problematic. On the negative side, the lack of separate sections or an intuitive index makes the discourse sometimes messy and disorganized forcing the reader to collect himself the relevant information from very different distant paragraphs.

Through the author's story we get to know, at the clinical level, how the internal functioning of her brain was affected [e.g., “*Do not think of obvious things*” (p. 33); “*Knocked out my decision-making abilities*” (p. 38); “*Some opposite words mixed up*” (before vs. after; yes vs. no, p. 51); “[did] *not have a sense of humor*” (p. 54); “*All bits of information seemed equally important*” (p.59); “[My memory lacked of temporal] *steps*” (p. 149)] as well as some subjective experiences of those symptoms (e.g., “*For some reason I found it easier to draw my copy directly beneath the doctor's prototype rather than alongside*” (p. 27); “*I had to see the “shampoo” I wanted to buy in my mind's eye*” (p.57); “[I found] *worse enlarged letters than regular size because the space in between was reduced*” (p. 89)]. Perhaps the most descriptive content is found at the social level, since the author makes us reconsider our frequent misbehavior toward stroke patients, specially highlighting the need to be treated as normal; the panic to be confronted in public if some error was committed [e.g., paying with large notes to avoid problems with the change; “*I vowed I would not go shopping again if that was the reception I would have to expect*” (p. 40)] and the tiredness to be so frequently interrupted that led her to an unfortunate change of leisure enjoyment toward non-linguistic requiring activities [e.g., “*It was a relief-the comforting darkness of the stadium and not having to talk to anybody*.” (p. 86)]. Moreover, the book is enlivened with some drawings that the author herself made to better explain some of these situations.

Free narrative of symptoms is not a new genre. One of the main success of Oliver Sacks was to become one of the first scientists to highlight the subjectivity of his patients, namely their individualized internal neurological functioning, rather than focusing on the objective damage (e.g., Sacks, 1985). Lucky us, Jennifer Gordon survived the stroke and recovered the ability of speech to give us fresh testimony and honest feedback equally useful for specialists and laymen. Under the eyes of specialists, the subjective experience of the illness would help with the theoretical description of the cognitive impairment and with the development of new therapies (e.g., the absence of “memory steps” reported by the author might inspire neuropsychologist to invent new recovery tasks). Under the eyes of laymen, this book would help to better understand the feelings of the patient and would provide ideas on how to make their day by day more manageable.

## Author contributions

The author confirms being the sole contributor of this work and approved it for publication.

### Conflict of interest statement

The author declares that the research was conducted in the absence of any commercial or financial relationships that could be construed as a potential conflict of interest.

## Footnotes

^1^Teasell et al. (2016).
